# Prediction of drug side effects with transductive matrix co-completion

**DOI:** 10.1093/bioinformatics/btad006

**Published:** 2023-01-19

**Authors:** Xujun Liang, Ying Fu, Lingzhi Qu, Pengfei Zhang, Yongheng Chen

**Affiliations:** NHC Key Laboratory of Cancer Proteomics, Department of Oncology; National Clinical Research Center for Gerontology, Xiangya Hospital, Central South University, Changsha 410008, China; NHC Key Laboratory of Cancer Proteomics, Department of Oncology; NHC Key Laboratory of Cancer Proteomics, Department of Oncology; NHC Key Laboratory of Cancer Proteomics, Department of Oncology; NHC Key Laboratory of Cancer Proteomics, Department of Oncology; National Clinical Research Center for Gerontology, Xiangya Hospital, Central South University, Changsha 410008, China

## Abstract

**Motivation:**

Side effects of drugs could cause severe health problems and the failure of drug development. Drug–target interactions are the basis for side effect production and are important for side effect prediction. However, the information on the known targets of drugs is incomplete. Furthermore, there could be also some missing data in the existing side effect profile of drugs. As a result, new methods are needed to deal with the missing features and missing labels in the problem of side effect prediction.

**Results:**

We propose a novel computational method based on transductive matrix co-completion and leverage the low-rank structure in the side effects and drug–target data. Positive-unlabelled learning is incorporated into the model to handle the impact of unobserved data. We also introduce graph regularization to integrate the drug chemical information for side effect prediction. We collect the data on side effects, drug targets, drug-associated proteins and drug chemical structures to train our model and test its performance for side effect prediction. The experiment results show that our method outperforms several other state-of-the-art methods under different scenarios. The case study and additional analysis illustrate that the proposed method could not only predict the side effects of drugs but also could infer the missing targets of drugs.

**Availability and implementation:**

The data and the code for the proposed method are available at https://github.com/LiangXujun/GTMCC.

**Supplementary information:**

Supplementary data are available at *Bioinformatics* online.

## 1 Introduction

Drugs could produce not only therapeutic effects but also some unexpected side effects. Side effects of drugs may have a strongly negative impact on human health. Usually, side effects are discovered by clinical trials or post-market surveillance. Some severe side effects will lead to the failure of drug development or the withdrawal of drugs from the market (Waring *et al.*, 2015; Wishart *et al.*, 2018). Thus, methods that could fast and economically identify potential side effects at the early stage of drug development are greatly needed.

Machine learning methods for predicting side effects could be a valuable complement to the costly traditional methods (Tan *et al.*, 2016). In recent years, researchers have advanced various machine learning methods which utilize different kinds of data to predict side effects. Chemical structures and protein targets are the most frequently used drug characteristics for side effect prediction (Nguyen *et al.*, 2021). For instance, Pauwels *et al.* (2011) used the chemical structures of drugs to predict side effects based on sparse canonical correlation analysis (SCCA). Mizutani *et al.* (2012) used the drug–target proteins as drug features and predicted side effects with SCCA. Iwata *et al.* (2013) applied $L_{1}$-regularized logistic regression to predict side effects and utilized target–protein domains as drug features. Other studies show that information integration could improve prediction performance. Liu *et al.* (2012) integrated the chemical substructures, protein targets, and phenotypic properties of drugs for side effect prediction. Based on the assumption that similar drugs tend to have similar side effects, Cao *et al.* (2015) combined multiple similarities calculated from different data sources, such as drug chemical structures and protein targets, to predict side effects. Zhang *et al.* (2018) proposed a method named feature-derived graph regularized matrix factorization (FGRMF). It projected the drug–side effect associations into low-rank space and utilized the graph regularization to incorporate the drug chemical and target similarities (Zhang *et al.*, 2018). Ding *et al.* (2019) developed a cosine similarity-based multiple kernel learning (MCS-MKL) algorithm to integrate information and improve prediction performance. In our previous work, we designed a Laplacian regularized sparse learning (LRSL) model to integrate multiple drug features for side effect prediction (Liang *et al.*, 2019). Additionally, to avoid manually designing the drug features and to explore the non-linear relationships in data, deep learning methods have been exploited to detect the side effects of drugs (Lee and Chen, 2021). For example, Zhao *et al.* (2021) developed a model for drug–side effect prediction based on the graph attention network to integrate different drug features.

Although the existing methods have shown considerable potential for the problem of side effect prediction, there are still some limitations of the previous studies. Firstly, side effects result from the perturbations of biological systems induced by the drug–target interactions. Thus, the knowledge of drug–target interactions is crucial for predicting and understanding side effects. Many existing methods utilize drug–target interactions as features to predict side effects (Nguyen *et al.*, 2021). However, the records for drug–target interactions are usually incomplete (Lomenick *et al.*, 2011). Many side effects are caused by the interactions between drugs and unintended off-targets (Lounkine *et al.*, 2012). Thus, the missing targets of drugs could deteriorate the predictive performance of the methods using protein targets as drug features. Secondly, as clinical trials usually include a selective and relatively small patient population, some side effects could only be discovered in the post-market stage (Noz *et al.*, 2019). Thus, these side effects are missing in the current knowledge of drugs. However, existing methods seldom deliberate on the influence of the missing side effects. In this study, we propose a novel side effect prediction algorithm that could deal with the problem of missing target proteins and the missing side effects in the task of side effect prediction. We formulate the side effect prediction as a multi-label learning problem with missing labels and features. The known drug–target associations and drug–side effect associations are represented as two binary matrices. Inspired by the previous studies (Goldberg *et al.*, 2010; Xu *et al.*, 2018), we assume that the drug–target matrix and the drug–side effect matrix are jointly low rank. We construct a predictive model based on matrix co-completion. Additionally, we suppose that only the positive relationships are observed in the drug–side effect matrix and the drug–target matrix, so we incorporate positive and unlabelled learning into our model. Finally, we take the graph constructed from the chemical similarities of drugs as an additional constraint for our model. The chemical structures, target proteins and side effects of drugs are collected. We test whether the proposed model could accurately predict side effects. Our model shows better predictive performance on side effect prediction compared with some state-of-the-art methods. The proposed model could also predict the missing targets of drugs. Thus, the proposed model could be an effective computational tool for predicting the side effects of drugs.

## 2 Methods

### 2.1 Data collection

The SIDER database records the side effects information of drugs (Kuhn *et al.*, 2016). We extracted the side effects of small molecular drugs from the SIDER database. We retrieved the chemical structures and human protein targets of drugs from the DrugBank database (Wishart *et al.*, 2018). We also retrieved the high-confidence protein–chemical interactions (the combined score>500) from the STITCH database (Szklarczyk *et al.*, 2016). Table 1 shows the summary of the collected data. It is noticed that 99.6% drug–target associations in the data from DrugBank, 98.7% drug–target associations in the data from Stitch and 93.8% drug–side effect associations are unobserved.

**Table 1.** The summary of our dataset

Data	Count
The number of drugs	962
The number of side effects	1877
The number of drug–side effect association	112 381
The number of fingerprints of drugs	881
The number of drug targets from DrugBank	1013
Number of drug targets from DrugBank and drug-associated proteins from STITCH	8833

The drugs that have known chemical structures, targets and side effects are kept for the following study. We represent the drug–side effect associations from SIDER as a binary matrix $Y\in {\left\{0,1\right\}}^{n\times c}$, where *n* is the number of drugs and *c* is the number of side effects. The elements Y(i,j)=1 if the *i*th drug is associated with the *j*th side effect, otherwise Y(i,j)=0. The side effects with less than five associated drugs were removed as it is difficult to train an effective model if the positive instances are too few. The drug–target associations extracted from DrugBank are represented as a binary matrix $X\in {\left\{0,1\right\}}^{n\times d}$, where *d* is the number of targets. The chemical structures of the drugs were extracted from DrugBank as SMILES strings. These SMILES strings were converted to the 881-bit fingerprints defined by PubChem using the R package ‘rcdk’ (Guha, 2007). The Tanimoto similarities between the fingerprint vectors were calculated to measure the chemical similarities between drugs. We represent the chemical similarities of drugs as a matrix $S\in R^{n\times n}$.

### 2.2 Problem formulation

In this work, the drug–target associations are denoted by *X*. The matrix *X* is divided into two sub-matrices $X_{\text{train}}\in R^{n_{\text{train}}\times d}$ and $X_{\text{test}}\in R^{n_{\text{test}}\times d}$, where $X_{\text{train}}$ is the training data which consists of $n_{\text{train}}$ drugs, $X_{\text{test}}$ is the test data which consists of $n_{\text{test}}$ drugs. The drug–side effect association matrix *Y* is also divided into $Y_{\text{train}}\in R^{n_{\text{train}}\times c}$ and $Y_{\text{test}}\in R^{n_{\text{test}}\times c}$, where $Y_{\text{test}}$ is for the test drugs that don’t have any known side effects (therefore, all elements in $Y_{\text{test}}$ are equal to zero). It should be noticed that some entries in *X* may be missing, as the records of the known drug–target associations are not complete. Also, there could be some missing elements in the matrix $Y_{\text{train}}$. Therefore, we treat the prediction of side effects as a multi-label transductive learning problem with missing features and labels in this study (Goldberg *et al.*, 2010).

In order to make the problem accessible, we assume that there is a linear dependence between *X* and *Y*. We also assume that *X* and *Y* are of low rank. As a result, the concatenated matrix $Z=[Y,X]\in R^{n\times (c+d)}$ is also of low rank. With the low-rank property, *Z* could be represented by

(1) $$\begin{array}{c} Z=W^{T}H \end{array},$$

where $W\in R^{t\times n}$, $H\in R^{t\times (c+d)}$ and t≪min(n,c+d). Consequently, we can impute the missing data in the concatenated matrix by solving the following problem:

(2) $$\begin{array}{c} \underset{W,H}{\min}\sum_{(i,j)\in \Omega }l(Z_{ij}-w_{i}^{T}h_{j})+r(W)+\tilde{r}(H) \end{array},$$

where Ω is the indices of the entries in *X* and $Y_{\text{train}}$. $Z_{ij}$ is the element in the *i*th row and *j*th column of *Z*. $w_{i}$ and $h_{j}$ are the columns of *W* and *H*. *l* is the loss function that calculates the difference between the predictive result $w_{i}^{T}h_{j}$ and the true value $Z_{ij}$. In this work, we use the squared error as the loss function. *r* and $\tilde{r}$ are the constraints of *W* and *H*, respectively.

As mentioned above, the feature matrix *X*, which denotes the drug–target associations, and the side-effect label matrix *Y* are both binary and have missing data. In practice, we only observe the positive associations between drugs and targets corresponding to 1 s in the matrix. It is also true for the drug–side effect associations in the matrix *Y*. The 0’s in *X* and *Y* are not negative entries, but signify that we don’t observe these values. Positive-unlabelled (PU) learning is the method for the situation where only a subset of positive entries are observed. In this study, we use the biased matrix completion proposed by Hsieh *et al.* (2015), which gives different weights (the hyper-parameter α) to the losses of positive entries and unobserved entries. According to Hsieh *et al.* (2015), minimizing the weighted loss in the partially observed situation is equivalent to minimizing the true recovery error. Therefore, we incorporate PU learning into our model. Due to the one-bit quantization and one-sided nature of the data, the first term in Equation (2) could be formulated as a biased matrix completion problem (Hsieh *et al.*, 2015)

(3) $$\begin{array}{c} \begin{array}{cc} & \underset{W,H}{\min}\sum_{(i,j)\in \Omega }\beta 1_{Z_{ij}=1}{\left(Z_{ij}-w_{i}^{T}h_{j}\right)}^{2}\\ & +\sum_{(i,j)\in \Omega }(1-\beta )1_{Z_{ij}=0}{\left(Z_{ij}-w_{i}^{T}h_{j}\right)}^{2}+r(W)+\tilde{r}(H) \end{array} \end{array},$$

where $1_{\cdot =1},1_{\cdot =0}$ are indicator functions. β∈(0,1) is the bias which gives different weights to observed and unobserved entries.

To integrate the chemical structure information of drugs into our model, we first construct a graph from the chemical similarity matrix *S*.

(4) $$\begin{array}{c} A_{ij}=\{\begin{array}{cc} S_{ij} & \;\text{if drug}\;i\;\text{and}\;j\;\text{are the}\;k-\text{nearest neighbours}\\ 0 & \text{otherwise} \end{array} \end{array}.$$

Let D=Diag(A1), we define the normalized graph Laplacian matrix as $L=D^{-\frac{1}{2}}(D-A)D^{-\frac{1}{2}}$. The matrix *W* is regularized by the chemical similarity graph:

(5) $$\begin{array}{c} \left\|w_{i}-w_{j}{\|}^{2}S_{ij}=\text{Tr}(WLW^{T}\right) \end{array}.$$

The above formula promotes the columns of *W* to be similar if the corresponding drugs have similar chemical structures. Then, formula (3) becomes

(6) $$\begin{array}{c} \begin{array}{cc} & \underset{W,H}{\min}\sum_{(i,j)\in \Omega }\beta 1_{Z_{ij}=1}{\left(Z_{ij}-w_{i}^{T}h_{j}\right)}^{2}\\ & +\sum_{(i,j)\in \Omega }(1-\beta )1_{Z_{ij}=0}{\left(Z_{ij}-w_{i}^{T}h_{j}\right)}^{2}\\ & +\gamma (WLW^{T})+\lambda_{1}\|W{\|}_{F}^{2}+\lambda_{2}\|H{\|}_{F}^{2} \end{array} \end{array},$$

where $\gamma ,\lambda_{1}$ and $\lambda_{2}$ are the hyper-parameters of our model. The constraint function $r(W)=\gamma (WLW^{T})+\lambda_{1}\|W{\|}_{F}^{2}$, while the constrain function $\tilde{r}(H)=\lambda_{2}\|H{\|}_{F}^{2}$. The additional $l_{2}$ regularization could assure convergence and reduce over-fitting. We name the above model as graph regularized transductive matrix co-completion (GTMCC).

### 2.3 Optimization

The objective function (6) could be solved by the proximal alternating linear minimization algorithm (Bolte *et al.*, 2014). The cost function without the constraint terms is

(7) $$\begin{array}{c} \begin{array}{cc} & f(W,H)=\sum_{(i,j)\in \Omega }\beta 1_{Z_{ij}=1}{\left(Z_{ij}-w_{i}^{T}h_{j}\right)}^{2}\\ & +\sum_{(i,j)\in \Omega }(1-\beta )1_{Z_{ij}=0}{\left(Z_{ij}-w_{i}^{T}h_{j}\right)}^{2} \end{array} \end{array}.$$

The partial gradient of f(W,H) with respect to *W* is $\nabla_{W}f(W,H)$. The partial gradient of f(W,H) with respect to *H* is $\nabla_{H}f(W,H)$. Let

(8) $$\begin{array}{c} G=W-\frac{1}{t_{w}}\nabla_{W}f(W,H) \end{array},$$

where $t_{w}$ is the step size. Then, we could update *W* by the proximal map

(9) $$\begin{array}{c} W_{k+1}={\text{prox}}_{t_{w}}^{r}(W_{k})=\text{argmin}\;r(W)+\frac{t_{w}}{2}\|W-G{\|}_{F}^{2} \end{array},$$

where $r(W)==\gamma (WLW^{T})+\lambda_{1}\|W{\|}_{F}^{2}$. Similarly, let

(10) $$\begin{array}{c} V=W-\frac{1}{t_{h}}\nabla_{H}f(W,H) \end{array},$$

*H* could also be updated by the proximal map

(11) $$\begin{array}{c} H_{k+1}={\text{prox}}_{t_{h}}^{\tilde{r}}(H_{k})=\text{argmin}\;\tilde{r}(H)+\frac{t_{h}}{2}\|H-V{\|}_{F}^{2} \end{array},$$

where $t_{h}$ is the step size and $\tilde{r}=\lambda_{2}\|H{\|}_{F}^{2}$. There are closed-form solutions for Equations (9) and (11). The step size $t_{w}$ and $t_{h}$ can be the Lipschitz constants of the corresponding partial gradients. Algorithm (1) outlines the optimal procedure of our model.


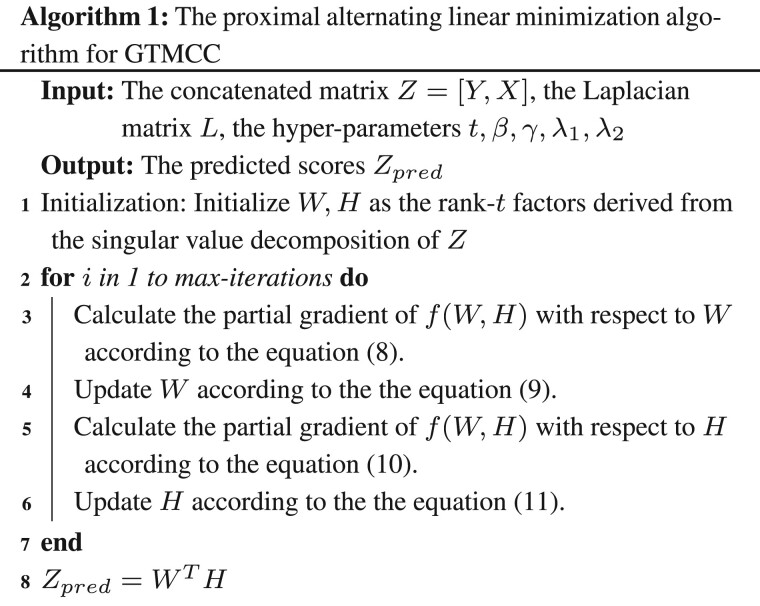


### 2.4 Model evaluation and comparison method

To illustrate the advantage of the proposed method, we compare it with several other side-effect prediction methods. Previous studies utilized SCCA to predict side effects with chemical structures or drug targets as the drug features (Mizutani *et al.*, 2012; Pauwels *et al.*, 2011). However, SCCA could not directly integrate multiple features of drugs. Thus, we compare the proposed method with sparse generalized canonical correlation analysis (SGCCA), which is an extension of SCCA for multi-view data. We implement SGCCA with the R package RGCCA (Tenenhaus and Guillemot, 2017). LRSL could integrate multi-view data with a graph regularized sparse linear regression model to predict side effects (Liang *et al.*, 2019). MCS-MKL fuses multiple drug-feature kernels by maximizing the cosine similarities between different kernels to predict side effects (Ding *et al.*, 2019). FGRMF is a method based on graph regularized matrix factorization (Zhang *et al.*, 2018). MGPred utilized the graph attention network to integrate different types of similarity information (Zhao *et al.*, 2021). The hyper-parameters of these comparison algorithms are determined by grid search and cross-validation. For SGCCA, 10 canonical components are kept, and the *L*1 penalization parameter c1 is set to 0.1. For LRSL, μ=0.1,β=0.01,λ=1,α=1,γ=2. For MCS-MK, μ=0.125,υ=0.0313. For FGRMF, k=80,μ=8,λ=4. For MGPred, the dropout coefficient is set to 0.5, the learning rate is 0.001 and the projection dimension is 64.

The performance of the proposed method and the comparison methods for side effect prediction are evaluated by 10-fold cross-validation experiments. In this study, we carry out two types of cross-validations. In the local cross-validation, all drugs in the collected dataset are split into ten subsets of roughly equal size. We mask all side effect labels of the drugs in one of these subsets each time. The remaining drug–side effect associations are used as training data. In the global cross-validation, all known drug–side effect associations are split into ten subsets. We use one subset of the drug–side effect associations as the testing data and take the remaining subsets as the training data. To get robust results, we repeat the cross-validation experiments ten times. We use five metrics to measure the performance of the comparison methods. AUC score is the area under the receiver operating characteristic curve. Average precision (AP) score is a summary of the precision–recall curve. AUC and AP scores are calculated globally by considering each element of $Y_{\text{test}}$ as a label. Label ranking AP (LRAP) score is a multi-label ranking metric based on the notion of label ranking. Coverage error (Co-error) is the average number of labels in the prediction results when all true labels are included. Ranking loss (Rloss) is the average of the number of label pairs that are incorrectly ordered. The higher values of AUC, AP and LRAP signify better performance, while the lower values of Co-error and Rloss indicate better performance. We use the implementations of these metrics in scikit-learn (Pedregosa *et al.*, 2012). Under the setting of the local cross-validation, the prediction could be regarded as a multi-label learning problem. All five metrics are calculated in the local cross-validation experiments. Because LRAP, Co-error and Rloss are specially designed for multi-label learning, only AUC and AP scores are calculated in the global cross-validation experiments.

## 3 Results

### 3.1 Parameter sensitivity analysis

**Fig. 1.**
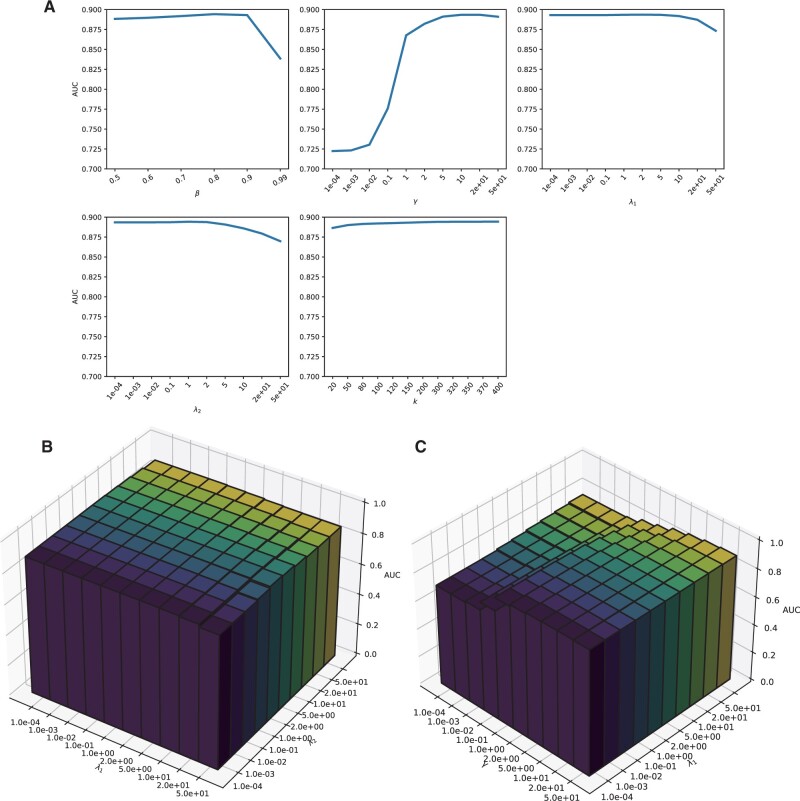
The influence of the hyper-parameters on the performance of the proposed method. (**A**). The influence of each single hyper-parameter on the predictive performance. (**B**). The influence of the combination of λ_1 _and λ_2 _on the performance. (**C**). The influnece of the combination of λ_1 _and Υ on the performance

There are five hyper-parameters in the proposed model, including $\beta ,\gamma ,\lambda_{1},\lambda_{2}$, and the dimension of the latent space *k*. Here, we examine the influences of these hyper-parameters on the performance of our model. We varied the values of each hyper-parameter and adopted 10-fold local cross-validation to evaluate the performance of the proposed model with the AUC score. The results are shown in Figure 1A. It is observed that the performance of the proposed method is most sensitive to the values of γ. When γ=10, the model achieves the best performance. When the value of γ decreases, the predictive performance also decreases. This result indicates that the chemical similarity information is important for our method to predict side effects. The second most influential hyper-parameter is β. This hyper-parameter introduces the label-dependent weights for the loss function. When β=0.8, our method performances best. Thus, the positive labels in the training data gain heavier weights. The result indicates that the biased matrix completion part in our model may relieve the adverse impact of data missing on the problem of side effect prediction. Next, we examined the influence of different combinations of $\lambda_{1}$ and $\lambda_{2}$ values. As shown in Figure 1B, although the predictive performance is not very sensitive to the combinations of $\lambda_{1}$ and $\lambda_{2}$ values when both of them are small, the AUC score decreases when the values of $\lambda_{1}$ and $\lambda_{2}$ are large. Thus, proper $l_{2}$ regularization of *W* and *H* plays an important role in our model. Additionally, as there could be possible interactions between the graph regularization term and the $l_{2}$ regularization of *W*, we examined the influence of different combinations of γ and $\lambda_{1}$ values. We find that the influence of γ value is dominant (Fig. 1C). At last, we set $k=400,\beta =0.8,\gamma =10,\lambda_{1}=2,\lambda_{2}=1$. These parameter values give the best AUC score and are adopted for the following analysis.

### 3.2 Comparison with other methods

#### 3.2.1 Results of the local cross-validation experiments

We compare the proposed method with three state-of-the-art methods for side effect prediction. All comparison methods in this study could integrate chemical and biological information of drugs. First, we evaluate the performance of different methods with 10-fold local cross-validation. As described in Section 2, all side-effect labels of the drugs in the testing dataset are masked in the local cross-validation experiments. This imitates predicting the side effects of novel drugs. We take the chemical substructures and the protein targets of drugs extracted from the DrugBank database as the drug features. In the 10-fold local cross-validation experiments, the proposed method shows the best performance in terms of all five metrics (Table 2). In AUC scores, the proposed method exceeds LRSL by 1.6%, MCS-MK by 3.0%, SGCCA and FGRMF by 3.5% and MGPred by 6.0%. For the side effect prediction problem, the positive labels are usually much less than the unknown/negative labels. As a result, AP and LRAP scores could be more sensitive metrics than the AUC score. In AP scores, the proposed method exceeds the second best-performed method LRSL by 3.5%. In LRAP scores, the proposed method exceeds the second best-performed method LRSL by 5%. Our method also shows improvement in Co-error and Rloss scores. The statistical results in Table 3 show that the improvement of the predictive performance is significant (*P*-value < 0.001, with the paired Wilcoxon test).

**Table 2.** The performance of the comparison methods using the protein targets from DrugBank in the 10-fold local cross-validation experiments

Method	AUC	AP	LRAP	Co-error	Rloss
SGCCA	0.8576±0.0076	0.3964±0.0169	0.4585±0.0172	1553.9948±45.2906	0.1166±0.0057
MCS-MK	0.8628±0.0080	0.3760±0.0292	0.4640±0.0177	1458.1310±46.0371	0.1104±0.0056
LRSL	0.8763±0.0067	0.4199±0.0177	0.4749±0.0168	1392.2091±43.7770	0.1001±0.0050
FGRMF	0.8577±0.0082	0.3782±0.0198	0.4183±0.0212	1523.5043±45.2615	0.1197±0.0084
MGPred	0.8322±0.0355	0.3237±0.0346	0.3585±0.0361	1556.6392±53.8307	0.1443±0.0701
GTMCC	0.8924±0.0082	0.4548±0.0272	0.5249±0.0198	1239.4647±49.7739	0.0841±0.0049

*Note*: The metrics are expressed as mean±standard deviation.

**Table 3.** The *P*-values of the comparison between GTMCC and the other methods with the paired Wilcoxon test using the protein targets from DrugBank in the 10-fold local cross-validation experiments

Method	*P*-value based on AUC	*P*-value based on AP	*P*-value based on LRAP	*P*-value based on Co-error	*P*-value based on Rloss
SGCCA	3.9559*e*−18	3.9559*e*−18	3.9559*e*−18	3.9559*e*−18	3.9559*e*−18
MCS-MK	3.9559*e*−18	3.9559*e*−18	3.9559*e*−18	3.9559*e*−18	3.9559*e*−18
LRSL	5.4074*e*−17	1.188*e*−14	4.8868*e*−18	4.3312*e*−18	4.3312*e*−18
FGRMF	3.9559*e*−18	3.9559*e*−18	3.9559*e*−18	3.9557*e*−18	3.9559*e*−18
MGPred	3.9559*e*−18	3.9559*e*−18	3.9559*e*−18	3.9559*e*−18	3.9559*e*−18

Next, we intend to include more drug–protein associations and examine whether the predictive performance could be improved. We extracted the high-confidence protein–chemical associations from the STITCH database and combined them with the drug–target data from DrugBank. After that, we utilized the expanded drug–protein interaction data to predict side effects and compared the performance of different methods. As shown in Table 4, the performance of the comparison methods increases when using the expanded drug–protein interactions data except for SGCCA. The proposed method still shows better performance than the other three methods. In AUC scores, the proposed method exceeds the second best method LRSL by 1.4%. In AP and LRAP scores, the proposed method exceeds LRSL by 5.9% and 5.8%, respectively. The proposed method also has better Co-error and Rloss scores. The differences in predictive performance between the proposed method and the other methods are significant (Table 5, *P*-value < 0.001).

**Table 4.** The performance of the comparison methods using the drug–protein associations from both DrugBank and STITCH in the 10-fold local cross-validation experiments

Method	AUC	AP	LRAP	Co-error	Rloss
SGCCA	0.8577±0.0077	0.4004±0.0166	0.4580±0.0174	1557.9269±46.0106	0.1172±0.0058
MCS-MK	0.8729±0.0069	0.3970±0.0307	0.4653±0.0180	1433.5795±43.7494	0.1093±0.0055
LRSL	0.8863±0.0070	0.4504±0.0229	0.4810±0.0175	1346.5484±41.4238	0.0983±0.0049
FGRMF	0.8577±0.0082	0.3821±0.0195	0.4258±0.0208	1523.4978±45.2636	0.1194±0.0084
MGPred	0.8492±0.0358	0.3485±0.0362	0.3685±0.0376	1543.9190±56.8017	0.1408±0.0861
GTMCC	0.9004±0.0083	0.5097±0.0295	0.5396±0.0215	1243.1037±50.0841	0.0832±0.0056

**Table 5.** The *P*-values of the comparison between GTMCC and other methods with the paired Wilcoxon test using the drug–protein associations from both DrugBank and STITCH in the 10-fold local cross-validation experiments

Method	*P*-value based on AUC	*P*-value based on AP	*P*-value based on LRAP	*P*-value based on Co-error	*P*-value based on Rloss
SGCCA	3.9559*e*−18	3.9559*e*−18	3.9559*e*−18	3.9559*e*−18	3.9559*e*−18
MCS-MK	3.9559*e*−18	3.9559*e*−18	3.9559*e*−18	3.9559*e*−18	3.9559*e*−18
LRSL	1.4921*e*−16	1.9899*e*−17	5.0364*e*−18	7.5593*e*−17	4.464*e*−18
FGRMF	3.9559*e*−18	3.9559*e*−18	3.9559*e*−18	3.9559*e*−18	3.9559*e*−18
MGPred	3.9559*e*−18	3.9559*e*−18	3.9559*e*−18	3.9559*e*−18	3.9559*e*−18

#### 3.2.2 Results of the global cross-validation experiments

In the global cross-validation experiments, 1/10 of the known drug–side effect associations are masked and the remained data are used for model training each time. It imitates predicting novel side effects of drugs that have known side-effect labels. Because the unknown drug–side effect associations (the zero elements in the matrix *Y*) are much more than the known ones, we randomly selected the same number of unknown drug–side effect associations as the negative samples. As a result, the ratio of negative to positive labels is 1 when calculating AUC and AP scores. First, only drug targets from DrugBank were used for prediction. The comparison results of the predictive performance are shown in Table 6. The proposed method shows the best performance among the comparison methods. It exceeds the second best method FGRMF by 2.5% in the AUC score and 2.3% in the AP score. The differences in predictive performance between the proposed method and the other method are significant (Table 6, *P*-value < 0.0001). We also combined the high-confidence protein–chemical associations from STITCH and the drug–target interactions from DrugBank to predict in the global cross-validation experiments. It is observed that the expended drug–protein associations don’t improve the performance of the proposed method in this situation (Table 7). But the proposed method still shows significantly better performance than the other method in both AUC and AP scores (Table 7, *P* < 0.001).

**Table 6.** The performance of the comparison methods and the *P*-values of the comparison between GTMCC and the other methods with the paired Wilcoxon test using the protein targets from DrugBank in the 10-fold global cross-validation experiments

Method	AUC	AP	*P*-value based on AUC	*P*-value based on AP
SGCCA	0.8565±0.0025	0.8596±0.0029	3.9559*e*−18	3.9559*e*−18
MCS-MK	0.9165±0.0018	0.9178±0.0019	3.9559*e*−18	3.9559*e*−18
LRSL	0.8765±0.0021	0.8757±0.0026	3.9559*e*−18	3.9559*e*−18
FGRMF	0.9324±0.0018	0.9380±0.0016	3.9559*e*−18	3.9559*e*−18
MGPred	0.8975±0.0420	0.9040±0.0416	3.9559*e*−18	3.9559*e*−18
GTMCC	0.9577±0.0013	0.9613±0.0012	–	–

**Table 7.** The performance of the comparison methods and the *P*-values of the comparison between GTMCC and the other methods with the paired Wilcoxon test using the drug–protein associations from both DrugBank and STITCH in the 10-fold global cross-validation experiments

Method	AUC	AP	*P*-value based on AUC	*P*-value based on AP
SGCCA	0.8558±0.0026	0.8600±0.0030	3.9559*e*−18	3.9559*e*−18
MCS-MK	0.9180±0.0018	0.9193±0.0019	3.9559*e*−18	3.9559*e*−18
LRSL	0.8869±0.0021	0.8867±0.0024	3.9559*e*−18	3.9559*e*−18
FGRMF	0.9117±0.0019	0.9185±0.0018	3.9559*e*−18	3.9559*e*−18
MGPred	0.8958±0.0567	0.9024±0.0576	3.9559*e*−18	3.9559*e*−18
GTMCC	0.9556±0.0013	0.9582±0.0013	–	–

### 3.3 Predicting side effects for novel drugs and inferring missing targets

To illustrate the application of our method, we predicted the side effects of the drugs from DrugBank which were not included in our dataset but had known targets or associated proteins. The number of these novel drugs is 3886. We trained our model with all drugs, the chemical structures, the target proteins and the associated proteins of the drugs and the known side effects. The predicted side effects for each drug are listed in Supplementary Table S1. We find that some prediction results could be validated by the literature. For example, flupentixol (DB00875) is a drug used for schizophrenia and depression. It has no record of side effects in our dataset. The top 1 ranked predicted side effect of butalbital is tachycardia (C0039231). This association has been proved by Karimi and Vahabzadeh (2014). Acemetacin (DB13783) is an anti-inflammatory drug for the treatment of pain and inflammation. The top 2 ranked predicted side effects by our method for it are diarrhoea (C0011991) and gastrointestinal haemorrhage (C0017181). These predicted side effects are supported by the literature Chandrasekharan (2007). For the side effect of hepatocellular injury (C0151763), the top 3 ranked predicted drugs are chlorpromazine (DB00477), ziprasidone (DB00246) and zileuton (DB00744). We find evidence to support the predicted results for chlorpromazine (Mitchell and Wilkinson, 1956) and zileuton (Watkins *et al.*, 2007).

Besides predicting side effects, our method could also predict the missing targets of drugs. To measure the target-prediction performance of the proposed method, we carried out 10-fold cross-validation experiments. For each fold, 10% known drug–protein associations were masked. We trained the model with all drug–side effect associations and the rest of the drug–protein associations. AUC and AP scores were calculated to evaluate the performance of target prediction. The average AUC score is 0.8874 and the average AP score is 0.9200 when only the targets from DrugBank are included. When the drug–protein associations from both DrugBank and STITCH are included, the average AUC score is 0.9273 and the average AP score is 0.9445. Therefore, the proposed method could also predict drug–protein associations accurately.

## 4 Discussion and conclusion

In this study, we propose a novel side effect prediction method. Specifically, we use the transductive matrix co-completion framework to handle the missing data in both the side-effect label matrix and the drug–target matrix. We concatenate these two matrices. Under the low-rank assumption, we explicitly represent the concatenated matrix using matrix factorization. Furthermore, because there are only a subset of positive entries observed in the concatenated matrix, we use the biased matrix completion method to give different weights to the observed and unobserved entries. Finally, we use graph regularization to incorporate the chemical information of drugs. Using cross-validation experiments, we show that the proposed method is significantly better than some state-of-the-art methods. Some prediction results could be confirmed by independent studies. Additionally, our method could also predict the targets of drugs.

Side effects are the results of the interactions between drugs and the biological system. Therefore, the targets of drugs are important for the prediction of side effects. However, the target information is incomplete for most drugs. In a previous study, Liu and Altman (2015) realized this problem and used protein structures to predict drug–target interactions before predicting side effects. But predicting every possible interaction between drugs and proteins in this way is very costly in time. On the other hand, side effects could be used to infer the targets of drugs as the phenotypic features of drugs (Campillos *et al.*, 2008). Therefore, we assume that the prediction of side effects and the imputation of drug targets will promote each other if we integrate these two problems into a model. Based on this idea, we propose a matrix co-completion method in this study. The experiment results support the assumption and show the proposed method performs better than some other methods.

For side effect prediction, only a subset of positive entries are observed and the true negative entries are not available. Thus, we incorporate PU learning into our model to handle the problem of learning from only positive and unlabelled entries. While the compared methods are not designed to deal with the unavailability of negative entries. Furthermore, our method is a transductive learning model, which could simultaneously explore the feature distribution of both the training and testing data to improve the prediction performance. Besides, we also keep some validated structures in previous models such as graph regularization and low-rank matrix completion in our model. All the above reasons could promote the prediction performance of our model.

Our method also has some limitations. First, the scope of drug targets in our method is limited to the known targets of drugs. As a result, if a drug causes a side effect by interacting with a protein target that is not in the known target space, our method could not reveal this association. In this work, when we expand the drug–target space by combining the drug-associated proteins from the STITCH database, the predictive performance is improved in the local cross-validation experiments (Tables 2 and 4). However, incorporating additional drug–protein associations may introduce noise to the data and the additional data could not improve the performance of our method in the global cross-validation experiments. Second, the proposed method is transductive. When predicting the side effects of novel drugs, we have to re-train the model with both testing data and training data. It will bring extra cost. Finally, our method could not illustrate the relationships between side effects and drug targets. Thus, the proposed method is less interpretable than LRSL. In the future, a method that is more robust to noise and more interpretable should be developed.

## Supplementary Material

btad006_Supplementary_Data
